# The value of QRS onset of the outflow tract PVC in V1 and V2 leads recorded in fourth, third, and second intercostal spaces to differentiate main origins of premature ventricular contraction—A prospective cohort study

**DOI:** 10.1002/hsr2.670

**Published:** 2022-06-16

**Authors:** Mohammad V. Jorat, Farzaneh Vaziri, Mani Hassanzadeh, Parsa Jorat, Zahra Mehdipour Namdar, Lobat Ataei Rooyani, Amir Aslani, Peyman Izadpanah

**Affiliations:** ^1^ Interventional Electrophysiologist Shiraz University of Medical Sciences Shiraz Iran; ^2^ Cardiology Department Shiraz University of Medical Sciences Shiraz Iran; ^3^ Shiraz University of Medical Sciences Shiraz Iran

**Keywords:** electrocardiography, intercostal spaces, PVC, start time

## Abstract

**Background:**

Electrocardiography (ECG) is now proposed as a simple and cost‐effective tool to determine the location of arrhythmias before ablation. We aimed to examine the value of the QRS onset of outflow tract PVC in V1 and V2 leads recorded in fourth, third, and second intercostal spaces to differentiate two main origins for premature ventricular contraction (PVC) including right ventricular outflow tract (RVOT) and left ventricular outflow tract (LVOT).

**Methods:**

In this prospective cohort study, a total of 58 patients were studied, from whom a surface ECG was obtained using V1 and V2 leads in the fourth, third, and second intercostal spaces. ECG and Electrophysiology studie (EPS) data were then recorded and compared to determine the sensitivity and specificity of QRS onset in locating arrhythmias. The reciever operating characterictic (ROC) curve analysis was applied to test diagnostic performance.

**Results:**

Based on the time of PVC initiation in each of the V1 and V2 leads in the fourth intercostal space, if PVC is recorded earlier in the V1 lead, its source in 95.8% of the patients is RVOT and if PVC preceded the V2 lead, 70.59% of the patients had PVC from LVOT. Comparing of QRS onset in V1 and V2 leadsrecorded from third% and and second intercostal spaces had considerable sensitivity and specificity to determine the origin of the outflow tract PVC (81.82 and 94.12%, respectively)

**Conclusion:**

Simultaneous recording of outflow tract PVCs from second third and fourth intercostal spaces and comparing their onset can determine the left and right outflow tract PVCs with high sensitivity and specificity.

**Abbreviations**
AC: aortic cusp
CAD: coronary artery disease
ECG: electrocardiography
LBBB: left bundle branch block
LVOT: left ventricular outflow tract
NSVT: nonsustained ventricular tachycardia
OTVA: outflow tract ventricular arrhythmia
PVC: premature ventricular contraction
RF: radio frequency; RFA, radio frequency ablation
RBBB: right bundle branch block
RVOT: right ventricular outflow tract
SMVT: sustained monomorphic ventricular tachycardia

## 1 INTRODUCTION

Premature ventricular contraction (PVC) is an extra heartbeat that originates in the ventricles.^1^ The outflow tract ventricular arrhythmia (OTVA) is the most common subtype of PVC of unknown origin that is now identified as a common cardiac complication, particularly in the elderly and those with cardiovascular risk profiles including hypertension and history of acute myocardial infarction; however, it is also observable in healthy and young people up to middle age and without even structural heart disease that can be triggered by psychological stress, exercise or food stimuli.^2^, ^3^ OTVAs can be clinically subclassified as paroxysmal sustained monomorphic ventricular tachycardia (SMVT), repetitive nonsustained ventricular tachycardia (NSVT), and PVCs. The prognosis of this arrhythmia is usually good and can be effectively treated with drugs or radiofrequency ablation (RFA).^4^, ^5^ Highly successful RFA is currently the preferred treatment for OTVA in symptomatic patients and/or in patients who do not respond to antiarrhythmic drugs (beta‐blockers or sodium channel blockers) as well as in patients with reduced left ventricular function induced by OTVA. Additionally, ablation procedures for OTVA vary depending on the origin of the arrhythmia. Therefore, differentiating the origin of OTVA based on electrocardiography (ECG) findings reduces treatment time and unnecessary punctures.^6^ PVC can have different origins. If the origin of PVC is in the right ventricle, it usually appears with the morphology of the left bundle branch block (LBBB) and if it is in the left ventricle, it usually appears with the view of the right bundle branch block (RBBB) because in each case, the involved ventricle depolarizes sooner. In general, three common zones are introduced as the origin of PVC including right ventricular outflow tract (RVOT) in 70%–80% of cases, left ventricular outflow tract (LVOT) in 10%–15%, and less commonly aortic cusp (AC).^7^, ^8^, ^9^ PVC originated from RVOT is more common in women between aged 30 and 50 years and usually occurs as a broad QRS complex with the LBBB pattern with inferior axis while PVC originated from LVOT is common in men older than 40 years and also in hypertensive patients with left ventricular dysfunction.^10^, ^11^, ^12^, ^13^, ^14^

Today, surface ECG is still one of the simplest and cost‐effective noninvasive methods for determining cardiac arrhythmias. By interpreting the appearance of ECG waves, it can be determined whether a heartbeat is related to sinus rhythm or is an arrhythmia.^15^ It is important to know the source of the PVC before starting the ablation operation. ECG interpretation is not only very helpful in preoperative planning for such arrhythmias, but it can potentially improve ablation results, reduce some procedural complications, reduce X‐ray exposure, decrease the duration of surgery and also reduce the risk of event recurrence.^16^, ^17^, ^18^, ^19^ Various algorithms and criteria based on surface ECG have been proposed to determine the location of an arrhythmia between the left and right ventricles. In the studies performed, the transition zone index (TZI), the length of R in V2, and R to S ratio in V2 had the highest value in this regard.^20^, ^21^

According to the importance of differentiating the location of PVC and its role in the treatment of patients, the present study aimed to compare the difference in the onset time of PVCs with left and right ventricular origin from thoracic leads taken in the second, third, and fourth intercostal spaces in the left and right lines adjacent to the sternum.

## 2 METHODS

This prospective cohort study was designed to determine the accuracy of a superficial ECG in diagnosing the origin of PVC, which was performed from September 2017 to March 2018 at the cardiovascular hospitals affiliated to Shiraz University of Medical Sciences.

The study was done on group of patients with frequent monomorphic PVC with morphology suggestive of outflow tract origin and arrhythmia burden more than 15% in 24 h. For this study, easy sampling method (availability sampling) was considered. Although few patients had less number but they were highly symptomatic.

Inclusion criteria were having informed consent to participate in the study, having asymptomatic PVC with PVC burden more than 15% of the total beats, having marked symptomatic PVC, or left ventricular dysfunction due to PVC and the presence of PVC originated from OT with LBBB morphology on surface ECG who underwent successful ablation. Those with unsuccessful ablation or recurrence of PVC within 24 h of ablation were excluded from the study. Ethically, given that the process of conducting this study was in line with the usual therapeutic interventions of patients and all patients needed EPS and ablation based on the decision of a cardiologist, so no intervention beyond what was in accordance with the therapeutic instructions was done. Informed consent was obtained from all patients or their guardians included in the study and a written consent form was completed.

## 3 DESCRIPTION OF THE ECG PROCEDURE

After obtaining informed consent from the patients, they were transferred to the cardiac electrophysiology laboratory in a fasting state. Under slight conscious sedation, using BARD EP recording system (Boston Scientific) simultaneous 12‐lead surface ECG and intracardiac recording was done by inserting a quadripolar 6F catheter (Irvine Biomedical) in the right ventricular apex. Mapping of the endocardial surface, coronary sinus, great cardiac vein, pulmonary artery, and aortic cusps was done by a 7F steerable catheter (Stinger). To find the best location of ablation in RVOT activation mapping and pace mapping were used and activation mapping was used to find the best location in other positions. The earliest activation site and/or the location with the best pace‐matched ECG configuration was used for finding site of the application of RF energy.

## 4 DESCRIPTION OF ECG RECORDING

**Figure 1:**
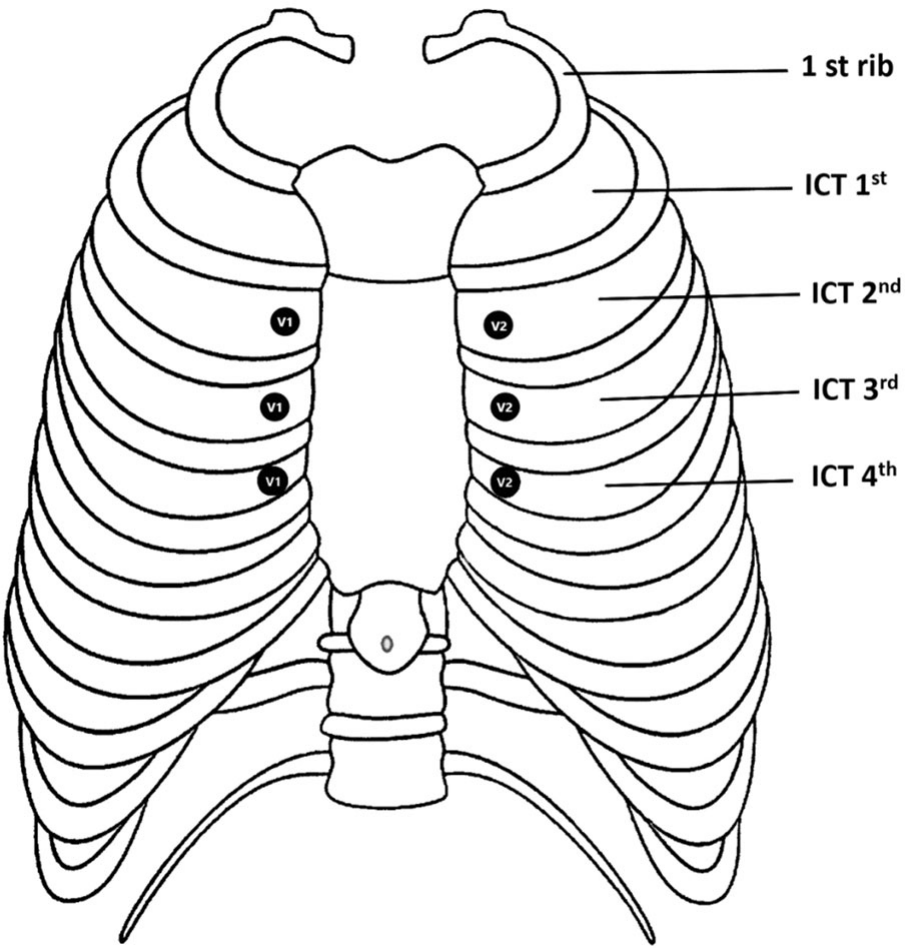
Position of V1 and V2 leads in the left and right parasternal borders in different spaces.

**Figure 2:**
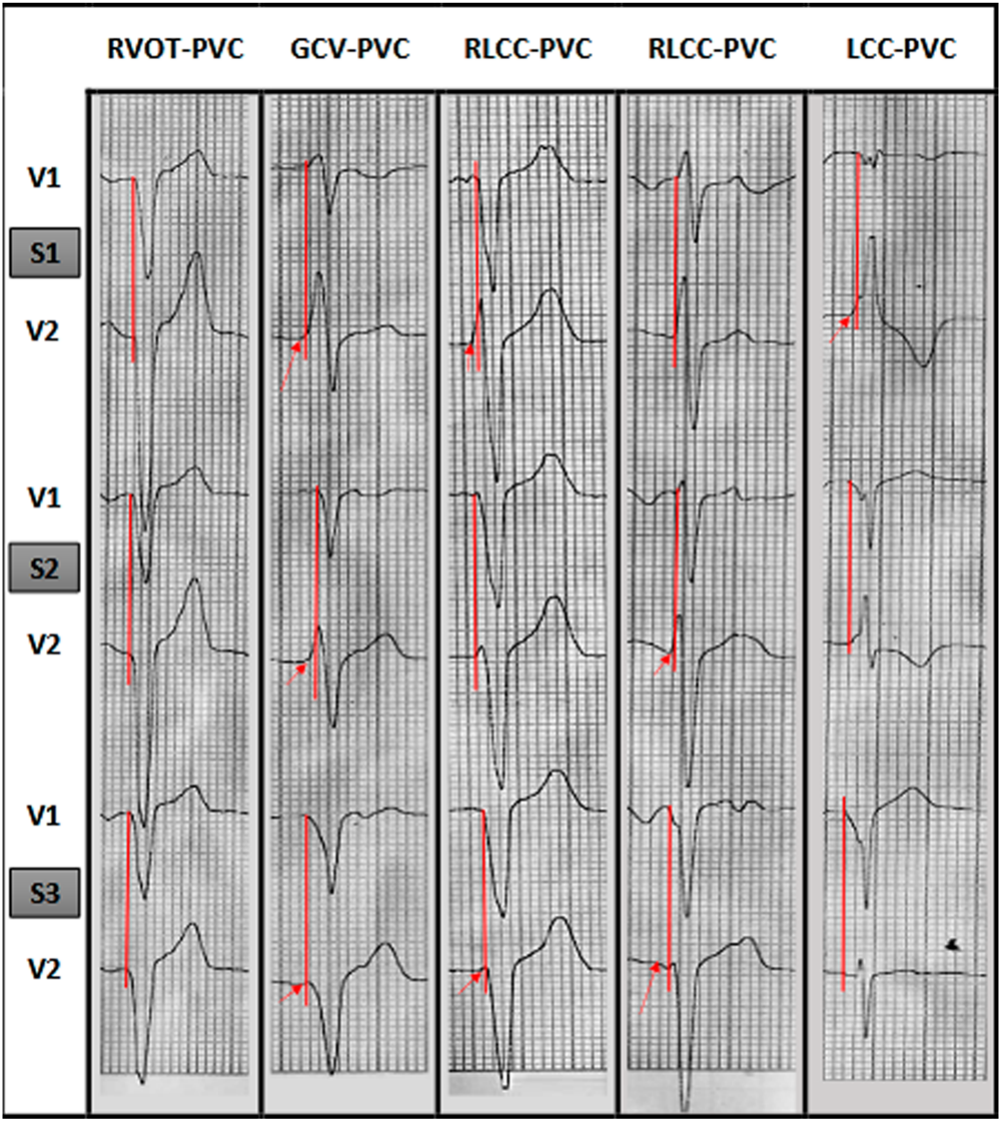
Premature ventricular contraction in intercostal spaces is shown in the given electrocardiography. The vertical line is drawn to compare QRS onset in positions V1 and V2 in the second, third and fourth spaces. The pointer shows the QRS start in V2 in each space.

Surface ECG was taken befre ablation. Regarding lead positioning, chest electrodes, V1 and V2 electrodes, were placed in the left and right parasternal lines in the fourth intercostal space (S1), V3 and V4 in the third intercostal space (S2), and V5 and V6 in the second intercostal space (S3), and the ECG of PVC wave was taken (Figures 1 and 2). Therefore, silmultaneous recording of PVC was done in these intercostal spaces while electrodes were located in V1 and V2 postions.

The ECGs were then reprinted at ×4 magnification. QRS initiation in PVC/VTs was marked on V1 and V2 leads. The time difference between the onset of QRS in VT at V1 and V2 leads in each of the intercostal spaces (S1, S2, and S3) was then measured using Scale Ruler 1:125, and the start time of the QRS wave at V1 and V2 were compared in each space. Each unit of the scale ruler was estimated at 0.008 s with a magnification of four times the above scale. ECG and EPS data were then recorded and compared in data collection forms to determine the sensitivity and specificity of ECG in locating arrhythmias. In this regard, data obtained from chest lead in the second, third, and fourth intercostal spaces on both sides of the sternum were compared with EPS findings.

To describe the data, descriptive statistics including mean and standard deviation for quantitative variables and number and frequency percentage for qualitative variables were used. The sensitivity and specificity of ECG to determine the zone of arrhythmia was assessed using the reciever operating characterictic (ROC) curve analysis and estimating area under the curve (AUC). In this regard, the AUC > 0.70 indicated an acceptable diagnostic value for ECG. For the statistical analysis, the statistical software SPSS version 23.0 for windows (IBM) was used.

## 5 RESULT

Fiftyeight patients fulfilled criteria of this study. Most of them did not have coronary artey disease; however, three of them were undergone coronary artery bypass surgery and two coronary stenting. Demographic data are seen in table 1, 2. From all of these patients studied, a surface ECG was obtained using V1 and V2 leads in the fourth intercostal space (S1). In 24 of these individuals, the onset of PVC in V1 was recorded earlier. By matching the EPS result, PVC originated from RVOT in 95.83% of the cases. In another 34 patients, the onset of PVC was recorded earlier in V2 lead that based on EPS findings, PVC was originated from LVOT in 70.59% of the patients.

To determine the value of comparing QRS onset in V1 and V2 ECG leads in intercostal fourth space (S1), third intercostal space (S2), and second intercostal space (S3), the differences of QRS onset and site of origin were were plotted and ROC curves was determined. It showed earlier QRS onset in V1 lead in S1 can predict RVOT origin of PVC. If PVC onset was earlier in V2, the sensitivity of the ECG to predict LVOT origin is not significant. However, when other data were added, they showed when QRS onset was earlier in V2 in both S1 and S3, it can predict accurately LVOT origin of PVC.

**Figure 3:**
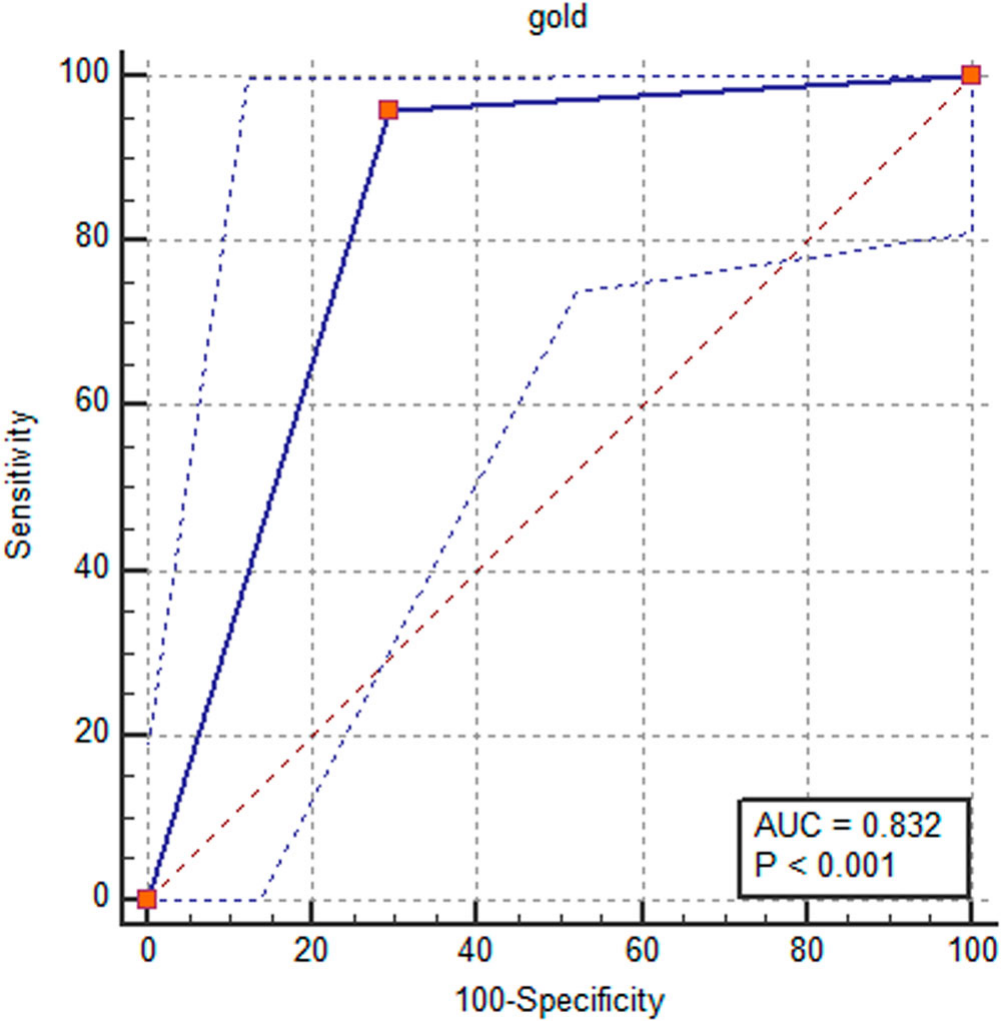
The ROC curve analysis to determine the value of V1 and V2 leads recorded in fourth intercostal space in determining the origin of premature ventricular contraction.

The pointed findings indicated high value of V1 and V2 leads recorded in the fourth intercostal space in determining the origin of PVC (AUC = 0.832, *p* < 0.001) with a sensitivity of 95.83% and a specificity of 70.59% (Figure 3).

**Table 1:** Demographic data of patients

Patients	Number	Male	Female	Age
RVOT	35	16	19	41.53 ± 11.43
LVOT	23	13	10	44.68 ± 12.63
Total	58	29	29	43.1 ± 12.03

*Note*: Age, average ± SD.

Abbreviations: LVOT, left ventricular outflow tract; RVOT, right ventricular outflow tract.

**Table 2:** Ejection fraction of patients

EF	40–59	3
	50–59	40
	≥60	15
	Average EF	53.72
	Standard deviation	5.23

Abbreviation: EF, ejection fraction.

**Figure 4:**
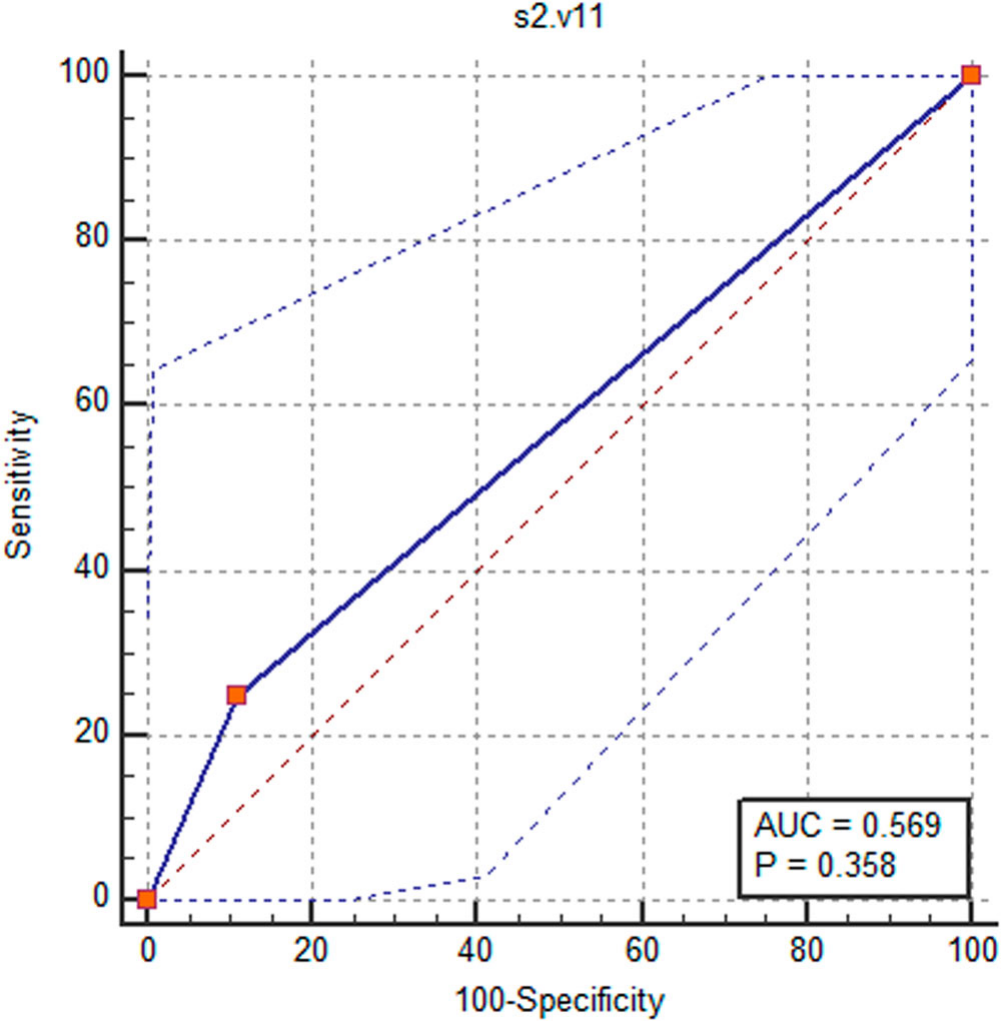
The ROC curve analysis to determine the value of V1 and V2 leads recorded in the third intercostal space in determining the origin of premature ventricular contraction.

A total of 30 patients with recorded PVC in V2 lead in the previous step and were re‐examined in the third intercostals space (S2). In 18 of these individuals, the onset of PVC in V2 was recorded earlier in S2 space and thus in 88.89% of patients, the PVCs recorded earlier in V2 lead originated from LVOT. The ROC curve analysis could not show the value of V1 and V2 leads recorded in third intercostal space to determine PVC source with sensitivity and specificity of 25.0% and 88.89%, respectively (Figure 4).

**Figure 5:**
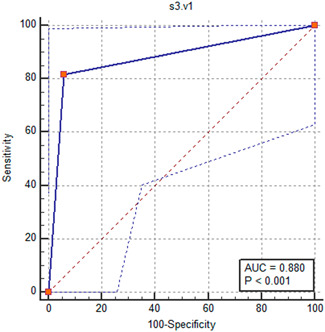
The ROC curve analysis to determine the value of V1 and V2 leads recorded in second intercostal space in determining the origin of premature ventricular contraction.

In total of 28 patients who had previously recorded PVC onset at V2 in the previous steps in both S1 and S2 spaces were re‐examined and onset of QRS was analysed in V1 and V2 leads in the second intercostal space (S3). In 17 of these individuals in S3 space, the onset of PVC in V2 lead was recorded earlier, and thus in 94.12% of cases of PVCs with earlier onset in V2 lead, the source of the arrhythmia was LVOT. Therefore, determining the zone of PVC at LVOT at S1, S2, S3 intercostal spaces was possible with an AUC of 0.880 (*p* < 0.001) yielding a sensitivity of 81.82% and a specificity of 94.12% (Figure 5).

In the next step, the analysis of surface ECGs in three S1, S2, and S3 spaces was done quantitatively and based on the priority when PVC was recorded in each of the leads and the algebraic sum of this priority and time delay in different spaces were calculated Among patients whose PVC was recorded earlier in V2 lead in S1 space and also the algebraic sum of time units in two higher spaces (S2 and S3) was more in V2 than V1, PVC was originated from LVOT. The sensitivity and specificity of this assessment to determine the place of PVC were 66.67% and 88.24%, respectively, with the ultimate AUC of 0.775 (*p* = 0.003).

## 6 DISCUSSION

PVC is one of the most common cardiac arrhythmias seen in patients with or without cardiac structural problems. A group of PVCs originate from RVOT and LVOT. Interventional treatment of this arrhythmia with ablation has a high success rate and few complications. Determining the location of PVC before intervention based on superficial ECG is important in preintervention planning and reduces complications, saves time and money, and minimizes X‐ray exposure in both patients and operators. Due to the fact that ECG is one of the simplest and most cost‐effective noninvasive methods for determining arrhythmias, ECG has been used in previous studies to diagnose the location of arrhythmias in various diseases such as Brugada syndrome.^20^, ^22^, ^23^, ^24^, ^25^ In this study, the sensitivity and specificity of using a surface ECG in V1 and V2 leads in the second, third, and fourth intercostal spaces were investigated to determine the location of PVC originating from OT. Based on the findings, a diagnostic algorithm can be defined to determine the location of PVC before ablation. Based on the priority of PVC initiation in each of the V1 and V2 leads in the fourth intercostal space, if PVC is recorded earlier in the V1 lead, its source in 95.8% of the patients is RVOT and if PVC preceded the V2 lead, 70.59% of the patients experience PVC from LVOT. In cases where PVC is recorded in V2 lead earlier, to increase the diagnostic accuracy, the ECG should be repeated it in the second and third intercostal spaces. Comparing the results obtained in two S2 and S3 spaces, it is revealed that if the beginning of PVC in V2 lead is observed earlier in S2 or S3 space, it can indicate the origin of PVC from LVOT with considerable sensitivity and specificity. Therefore, it is suggested that this spaces be evaluated after S1 space. It is also possible to explain the quantitative algorithm based on the time difference of PVC start of V1 and V2 leads in different spaces by using PVC start time priority in the pointed leads. In this regard, after taking ECG in S1 space from V1 and V2 leads, if PVC is recorded in V1 lead earlier, PVC originates from RVOT in 95.8% of patients, and in case of PVC in V2 lead, from LVOT in 70.59% of patients. In cases where PVC is recorded in V2 lead earlier, to increase the diagnostic accuracy, the ECG is repeated in the second and third intercostal spaces. We measure and compare the time difference of PVC priority in each of the leads in these two spaces. If the algebraic sum of the temporal precedence in the two higher spaces is superior in V2, we can consider the origin of LVOT for PVC with high sensitivity and specificity. Therefore, the use of PVC QRS start at V1 and V2 leads in the fourth, third, and second intercostal spaces can be determined the source of PVC arrhythmia (LVOT or RVOT) with significant sensitivity and specificity.

Some studies have attempted to simplify discovering the source of PVC by using ECG, however, contradictory results have been reported with respect to the diagnostic accuracy of ECG because a variety of ECG parameters have been introduced, but none of the parameters had enough accuracy to focally point to the origin of the arrhythmia. In a study by Ge et al.,^17^ patients with PVC/VT with LVOT origin were evaluated and compared to those with PVCs originated from RVOT. In their study, if the chest lead transition was seen in pre‐V3 leads and the TZI of the chest leads was greater than 0, the PVC was sourced from LVOT origin. In another study by Kimie et al.,^26^ V2 transition ratio was significantly higher in PVCs originated from LVOT group than from RVOT and V2 transition ratio could predict LVOT origin with sensitivity of 83% and specificity of 88%. Efimova et al.^8^ examined the time interval between the onset of the earliest QRS complex in PVC and the right ventricular apical signal in patients referred for ablation. In patients with PVCs originated from LVOT, the QRS‐RVA interval was significantly longer than in patients with PVC sourced from RVOT origin and thus QRS‐RVA interval of more than 49 ms could predict LVOT as the origin for PVC with sensitivity, specificity, positive and negative predictive values of 94.7%, 95.0%, 95.0%, and 94.7%, respectively. Thus, various ECG‐based criteria have been introduced to predict the origin of PVC. It seems that by combining these criteria and achieving the highest diagnostic performance, it is possible to easily distinguish the two main sources for this arrhythmia (LVOT from RVOT) and, therefore the efficiency of ablation for eliminating PVCs can be maximized.

## 7 CONCLUSION

According to present study, it can be concluded that by using some ECG‐based criteria including the priority of PVC initiation in each of the V1 and V2 leads in the second, third, and fourth intercostal spaces, the differentiation of the two sources for PVCs (LVOT and RVOT) can be possible and thus the efficacy of ablation to eliminate such arrhythmia can be optimized.

## 8 LIMITATIONS

This study was performed mostly on patients with normal hearts, and it may not be generalized to patients with structural heart disease. It can be attributable to the fact that the mechanism of arrhythmia in patients with normal heart is different from that of patients with structural heart disease (focal triggered activity vs. scar related).

## AUTHOR CONTRIBUTIONS

**Mohammad V. Jorat**: Conceptualization; data curation; methodology. **Farzaneh Vaziri**: Data curation; project administration. **Mani Hassanzadeh**: Conceptualization; writing—review and editing. **Parsa Jorat**: Formal analysis. **Zahra M. Namdar**: Writing—original draft. **Lobat A. Rooyani**: Writing—review and editing. **Amir Aslani**: Formal analysis; project administration. **Peyman Izadpanah**: Writing—original draft. All authors have read and approved the final version of the manuscript. Professor Mani Hassanzadeh had full access to all of the data in this study and takes complete responsibility for the integrity of the data and the accuracy of the data analysis.

## CONFLICT OF INTEREST

The authors declare no conflict of interest.

## TRANSPARENCY STATEMENT

Professor Mani Hassanzadeh affirms that this manuscript is an honest, accurate, and transparent account of the study being reported; that no important aspects of the study have been omitted; and that any discrepancies from the study as planned (and, if relevant, registered) have been explained

## Data Availability

The data that support the findings of this study are available from the corresponding author upon reasonable request.

## References

[1] Pascale P , Pruvot E , Graf D , Metzger J , Fromer M , Schläpfer J . Idiopathic premature ventricular complexes originating from the ventricular outflow tract: evaluation, prognosis and management. Rev Med Suisse. 2010;6(1140):1142‐1145. 20572358

[2] John RM , Stevenson WG . Outflow tract premature ventricular contractions and ventricular tachycardia: the typical and the challenging. Card Electrophysiol Clin. 2016;8:545‐554. 27521088 10.1016/j.ccep.2016.04.004

[3] Korshunov V , Penela D , Linhart M , et al. Prediction of premature ventricular complex origin in left vs. right ventricular outflow tract: a novel anatomical imaging approach. Europace. 2019;21:147‐153. 30016418 10.1093/europace/euy162

[4] Benhayon D , Nof E , Chik WW , Marchlinski F . Catheter ablation in the right ventricular outflow tract associated with occlusion of left anterior descending coronary artery. J Cardiovasc Electrophysiol. 2017;28:347‐350. 27885742 10.1111/jce.13130

[5] Calvo N , Jongbloed M , Zeppenfeld K . Radiofrequency catheter ablation of idiopathic right ventricular outflow tract arrhythmias. Indian Pacing Electrophysiol J. 2013;13:14‐33. 23329871 10.1016/s0972-6292(16)30585-x PMC3540113

[6] Jiao, Z , Li Y , Mao J , et al. Differentiating origins of outflow tract ventricular arrhythmias: a comparison of three different electrocardiographic algorithms. Braz J Med Biol Res. 2016;49:49. 10.1590/1414-431X20165206 PMC4855996 27143173

[7] Dixit S , Gerstenfeld EP , Callans DJ , Marchlinski FE . Electrocardiographic patterns of superior right ventricular outflow tract tachycardias: distinguishing septal and free‐wall sites of origin. J Cardiovasc Electrophysiol. 2003;14:1‐7. 12625602 10.1046/j.1540-8167.2003.02404.x

[8] Efimova E , Dinov B , Acou W‐J , et al. Differentiating the origin of outflow tract ventricular arrhythmia using a simple, novel approach. Heart Rhythm. 2015;12:1534‐1540. 25847476 10.1016/j.hrthm.2015.04.004

[9] Ouyang F , Fotuhi P , Ho SY , et al. Repetitive monomorphic ventricular tachycardia originating from the aortic sinus cusp: electrocardiographic characterization for guiding catheter ablation. J Am Coll Cardiol. 2002;39:500‐508. 11823089 10.1016/s0735-1097(01)01767-3

[10] Asirvatham SJ . Correlative anatomy for the invasive electrophysiologist: outflow tract and supravalvar arrhythmia. J Cardiovasc Electrophysiol. 2009;20:955‐968. 19490263 10.1111/j.1540-8167.2009.01472.x

[11] Dong X , Tang M , Sun Q , Zhang S . Anatomical relevance of ablation to the pulmonary artery root: clinical implications for characterizing the pulmonary sinus of valsalva and coronary artery. J Cardiovasc Electrophysiol. 2018;29:1230‐1237. 29978934 10.1111/jce.13685

[12] Kamioka M , Mathew S , Lin T , et al. Electrophysiological and electrocardiographic predictors of ventricular arrhythmias originating from the left ventricular outflow tract within and below the coronary sinus cusps. Clin Res Cardiol. 2015;104:544‐554. 25633492 10.1007/s00392-015-0817-4

[13] Kumagai K . Idiopathic ventricular arrhythmias arising from the left ventricular outflow tract: tips and tricks. J Arrhythm. 2014;30:211‐221.

[14] Viswanathan K , Mantziari L , Butcher C , et al. Evaluation of a novel high‐resolution mapping system for catheter ablation of ventricular arrhythmias. Heart Rhythm. 2017;14:176‐183. 27867071 10.1016/j.hrthm.2016.11.018

[15] Sirichand S , Killu AM , Padmanabhan D , et al. Incidence of idiopathic ventricular arrhythmias: a population‐based study. Circ Arrhythm Electrophysiol. 2017;10:e004662. 28183845 10.1161/CIRCEP.116.004662 PMC5319731

[16] Bala R , Marchlinski FE . Electrocardiographic recognition and ablation of outflow tract ventricular tachycardia. Heart Rhythm. 2007;4:366‐370. 17341405 10.1016/j.hrthm.2006.11.012

[17] Ge B , Ji K‐T , Ye H‐G , et al. Electrocardiogram features of premature ventricular contractions/ventricular tachycardia originating from the left ventricular outflow tract and the treatment outcome of radiofrequency catheter ablation. BMC Cardiovasc Disord. 2012;12:1‐11. 23186541 10.1186/1471-2261-12-112 PMC3571934

[18] Park KM , Kim YH , Marchlinski FE . Using the surface electrocardiogram to localize the origin of idiopathic ventricular tachycardia. Pacing Clin Electrophysiol. 2012;35:1516‐1527. 22897344 10.1111/j.1540-8159.2012.03488.x

[19] Zhang F , Hamon D , Fang Z , et al. Value of a posterior electrocardiographic lead for localization of ventricular outflow tract arrhythmias: the V4/V8 ratio. JACC Clin Electrophysiol. 2017;3:678‐686. 29759536 10.1016/j.jacep.2016.12.018

[20] Anderson RD , Kumar S , Parameswaran R , et al. Differentiating right‐and left‐sided outflow tract ventricular arrhythmias: classical ECG signatures and prediction algorithms. Circ Arrhythm Electrophysiol. 2019;12:007392. 10.1161/CIRCEP.119.007392 31159581

[21] Hachiya H , Aonuma K , Yamauchi Y , et al. Electrocardiographic characteristics of left ventricular outflow tract tachycardia. Pacing Clin Electrophysiol. 2000;23:1930‐1934. 11139960 10.1111/j.1540-8159.2000.tb07055.x

[22] Enriquez A , Baranchuk A , Briceno D , Saenz L , Garcia F . How to use the 12‐lead ECG to predict the site of origin of idiopathic ventricular arrhythmias. Heart Rhythm. 2019;16:1538‐1544. 30954600 10.1016/j.hrthm.2019.04.002

[23] Enriquez A , Malavassi F , Saenz LC , et al. How to map and ablate left ventricular summit arrhythmias. Heart Rhythm. 2017;14:141‐148. 27664373 10.1016/j.hrthm.2016.09.018

[24] Miyamoto K , Yokokawa M , Tanaka K , et al. Diagnostic and prognostic value of a type 1 Brugada electrocardiogram at higher (third or second) V1 to V2 recording in men with Brugada syndrome. Am J Cardiol. 2007;99:53‐57. 17196462 10.1016/j.amjcard.2006.07.062

[25] Nagase S , Hiramatsu S , Morita H , et al. Electroanatomical correlation of repolarization abnormalities in Brugada syndrome: detection of type 1 electrocardiogram in the right ventricular outflow tract. J Am Coll Cardiol. 2010;56:2143‐2145. 21144977 10.1016/j.jacc.2010.06.050

[26] Kimie O , Ichiro W , Yasuo O , et al. ECG criteria for distinguishing left from right ventricular outflow tract tachycardia. J Nihon Univ Med Assoc. 2015;74:95‐102.

